# Concept and Evaluation of a New Piezoelectric Transducer for an Implantable Middle Ear Hearing Device

**DOI:** 10.3390/s17112515

**Published:** 2017-11-02

**Authors:** Houguang Liu, Jinlei Cheng, Jianhua Yang, Zhushi Rao, Gang Cheng, Shanguo Yang, Xinsheng Huang, Mengli Wang

**Affiliations:** 1 School of Mechatronic Engineering, China University of Mining and Technology, Xuzhou 221116, China; liuhg@cumt.edu.cn (H.L.); jianhuayang@cumt.edu.cn (J.Y.); chg@cumt.edu.cn (G.C.); Ysgcumt@cumt.edu.cn (S.Y.); wangmengli@cumt.edu.cn (M.W.); 2 State Key Laboratory of Mechanical System and Vibrations, Shanghai Jiaotong University, Shanghai 200240, China; Jinlei.cheng@faw-vw.com; 3 Department of Otorhinolaryngology, Zhongshan Hospital affiliated to Fudan University, Shanghai 200032, China; huang.xinsheng@zs-hospital.sh.cn

**Keywords:** implantable middle ear hearing device, hearing loss, piezoelectric transducer, piezoelectric stack, incus body, temporal bone, evaluation studies

## Abstract

Implantable middle ear hearing devices (IMEHDs) have been developed as a new technology to overcome the limitations of conventional hearing aids. The piezoelectric cantilever transducers currently used in the IMEHDs have the advantages of low power consumption and ease of fabrication, but generate less high-frequency output. To address this problem, we proposed and designed a new piezoelectric transducer based on a piezoelectric stack for the IMEHD. This new transducer, attached to the incus body with a coupling rod, stimulates the ossicular chain in response to the expansion-and-contraction of its piezoelectric stack. To test its feasibility for hearing loss compensation, a bench testing of the transducer prototype and a temporal bone experiment were conducted, respectively. Bench testing results showed that the new transducer did have a broad frequency bandwidth. Besides, the transducer was found to have a low total harmonic distortion (<0.75%) in all frequencies, and small release time (1 ms). The temporal bone experiment further proved that the transducer has the capability to produce sufficient vibrations to compensate for severe sensorineural hearing loss, especially at high frequencies. This property benefits the treatment of the most common sloping high-frequency sensorineural hearing loss. To produce a 100 dB SPL equivalent sound pressure at 1 kHz, its power consumption is 0.49 mW, which is low enough for the transducer to be utilized in the IMEHD.

## 1. Introduction

Hearing loss is a major health problem affecting a very large portion of the general population and has a very wide range of etiologies [1]. In the aging population, 31% of individuals between the ages of 60 and 69 and 63.1% of those aged 70 years and older are hearing impaired [2]. Overall, approximately 360 million people worldwide have hearing loss. In terms of which part of the auditory system is affected, hearing loss can be classified into two types: conductive hearing loss and sensorineural hearing loss. With the development of ear microsurgical techniques, most of the conductive hearing loss can benefit from surgical interventions. However, there is still a lack of effective treatments for sensorineural hearing loss. The majority of these hearing-impaired individuals can only turn to conventional acoustic hearing aids that amplify sounds to compensate for the decrease in hearing sensitivity [3]. However, conventional hearing aids have several inherent disadvantages, such as sound distortion, limited amplification, feedback annoyance, ear canal discomfort, and stigma of wearing an external appliance [4], and 24% of the hearing aids owners cease to use them [5]. To solve these problems, many institutions began to investigate the implantable middle ear hearing devices (IMEHDs). Different from conventional hearing aids, which operate by overdriving the eardrum with their loudspeaker’s acoustic energy, IMEHDs compensate hearing loss by their implanted transducers’ mechanical stimulation to the ossicles (i.e., malleus, incus, stapes) [6,7,8], eardrum [9,10,11], or round window [12,13,14,15]. This IMEHDs’ mechanical simulation eliminates the acoustic feedback problem of hearing aids and increases the sound’s fidelity [16]. Besides, because the IMEHDs have their transducer implanted in the human body and leave the ear canal completely open, they overcome the problem of ear canal occlusion and improve patients’ comfort. Clinical report also shows that IMEHDs have much better high-frequency hearing gain and superior word recognition score than hearing aids [17].

From 1990, several types of IMEHDs have been investigated or developed in the world [16]. Among them, the incus-body driving type IMEHD, which has its transducer attached to the incus body, is one of the widely investigated types for its minor damage to the structure of the middle ear system [18,19,20] and less side effects on patient’s high-frequency residual hearing [21]. Up to now, this type of IMEHD uses either an electromagnetic transducer or a piezoelectric cantilever transducer as its vibrator. Comparatively, the piezoelectric cantilever transducer has demonstrated many advantages including ease of fabrication, lower power consumption, and compatibility with external magnetic fields. However, it cannot generate sufficient stimulating displacement at high frequencies owing to its specific structure [22]. This less high-frequency output makes it inadequate for its treatment since the most common type of sensorineural hearing loss is high frequency hearing loss [3].

To overcome the deficiency of the piezoelectric cantilever transducer, Wang et al. [22] proposed using a piezoelectric stack to realize the IMEHD’s piezoelectric transducer. They carried out a temporal bone experiment with a piezoelectric stack glued to the incus body by epoxy resin. Their experimental results show that the piezoelectric stack did have a better hearing loss compensation performance at high frequencies. To further study the influence of the piezoelectric stack parameters on the hearing loss compensating performance, we constructed a coupling mechanical model of the transducer and the middle ear [23]. Our model-predicted and experimental results demonstrate that stapes displacement stimulated by a 50 layers Lead Zirconate Titanate (PZT) piezoelectric stack excitation at 10.5 V RMS was equivalent to that from acoustic stimulation at 100 dB SPL. However, both of our and Wang et al.’s reports just proved the advantages of the piezoelectric stack to compensate hearing loss. None of these studies designed a piezoelectric stack transducer that can be implanted.

Accordingly, in this paper, based on our previous theoretical research and ear anatomy, a new piezoelectric transducer using the piezoelectric stack was designed for the incus-body driving type IMEHD. Finally, a prototype of this transducer was fabricated and its fundamental properties were evaluated by a bench testing and a temporal bone experiment. The experimental results showed that this transducer does have good performance at high frequencies, which benefits its application in patients with sensorineural hearing loss.

## 2. Materials and Methods

### 2.1. Device Design and Fabrication

**Figure 1:**
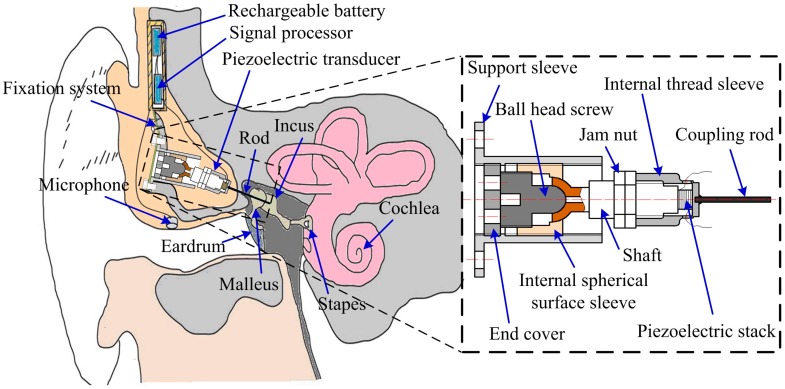
Illustration of the proposed implantable middle ear hearing device and its piezoelectric transducer.

The proposed IMEHD is schematically illustrated in Figure 1. This IMEHD includes a microphone for receiving an acoustic signal from outside, a signal processor for receiving and processing the signal from the microphone to generate a driving electrical signal, a piezoelectric transducer for generating a vibration in response to the driving electrical signal from the signal processor, and a rechargeable battery for powering this system. During surgery, the piezoelectric transducer is implanted into the mastoid cavity and held by a fixation system. Meanwhile, the output side of the transducer is attached to the incus body through a coupling rod.

Moreover, the inner structure of the designed piezoelectric transducer is also shown in Figure 1. This transducer consisted of a piezoelectric stack, a coupling rod, an internal thread sleeve, a shaft, two nuts, a support sleeve, an internal spherical surface sleeve, a ball head screw, and an end cover. Among them, the piezoelectric stack, which is a monolithic ceramic construction of many thin piezoelectric ceramic layers, is a key component since it generates the mechanical vibration for the transducer. The piezoelectric stack is enclosed in the internal thread sleeve. One side of the internal thread sleeve is held to the shaft, while the other side is stuck to the coupling rod, which is attached to the incus body. Thus, the incus body can be stimulated by the internal thread sleeve’s elastic deformation, which is caused by the repetition of the piezoelectric stack’s expansion and contraction in response to the applied voltage. Besides, to facilitate the axial adjustment and the radial adjustment of the coupling rod’s tip, a screw thread pair between the support sleeve and the internal spherical surface sleeve, and a spherical pair between the spherical shaft and the ball head screw were designed, respectively. The detailed explanation of this adjustment was given in Section 3.1.

The maximum outer diameter of the piezoelectric transducer is set to 10 mm, considering a 10–12 mm width hole can be drilled in the mastoid [24]. The diameter of the coupling rod is 1 mm. The piezoelectric stack PL022.30, which was fabricated by Physik Instrumente, Germany, was chosen for this transducer according to the results calculated by our previous human middle ear finite element model [23]. Specifically, the piezoelectric stack has a 2 × 2 mm^2^ rectangular cross section with a thickness of 2 mm. The piezoelectric stack’s material is Lead Zirconate Titanate (PZT) ceramics, considering its common use and low cost. The layer number of the piezoelectric stack is 50.

### 2.2. Vibration Displacement of the Piezoelectric Transducer

Transducer’s vibration displacement is directly related to the IMEHD’s hearing compensating performance. To ensure the transducer has the capacity to compensate severe sensorineural hearing loss, unloaded displacement of this transducer was measured firstly. We fixed the transducer’s support sleeve to an anti-vibration table by a magnetic base and used a laser vibrometer (OFV-505 sensor head, OFV-5000 vibrometer controller, Polytec, Waldbronn, Germany) to measure the displacement of the coupling rod of the transducer driven by a sine wave voltage with a frequency range from 200 Hz to 9 kHz. The driving voltage of the transducer was generated by a function generator (AFG3000 series, Tektronix, Beaverton, OR, USA). The measurement results of the laser vibrometer were collected by an LMS SCADAS data acquisition system.

In addition to the requirements of sufficient displacement and wide bandwidth, the transient dynamic behavior of an IMEHD’s transducer plays an equally important role for the degree of transmission quality that can be achieved. This aspect is particularly important for faithfully reproducing a complex sound signal [25]. To determine this transient dynamic behavior, the transducer was excited with a very short voltage pulse. The amplitude and the duration of the applied voltage pulse were 12 V_p-p_ and 50 μs, respectively, and the resulting mechanical response of the transducer was measured by the laser vibrometer.

### 2.3. Distortion Analysis of the Transducer

Low nonlinear distortions play a significant role in producing high-fidelity sound, especially for speech and music perception [25]. The total harmonic distortion (THD) is one of the common measures of nonlinear distortion and was utilized by many researchers to assess the implantable middle ear hearing devices’ distortions [7,22,26]. To derive the THD of this piezoelectric transducer, we used the function generator (Tektronix AFG3000) to apply a set of single-frequency voltages to the transducer, respectively. Their corresponding amplitude-frequency response curves of the transducer’s displacements were obtained by the Laser vibrometer. Based on these amplitude-frequency response curves, the THD of this transducer at this set of frequencies can be calculated according to Equation (1), relatively.

(1) $$THD=\sqrt{\frac{1}{A_{1}^{2}}\sum_{n=2}^{N}A_{n}^{2}}$$

where, *A*_1_ is the transducer displacement’s amplitude of the first harmonic frequency, *A_n_* is the amplitude of the nth harmonic frequency, and *N* is the total number of harmonics.

### 2.4. Temporal Bone Experiment

**Figure 2:**
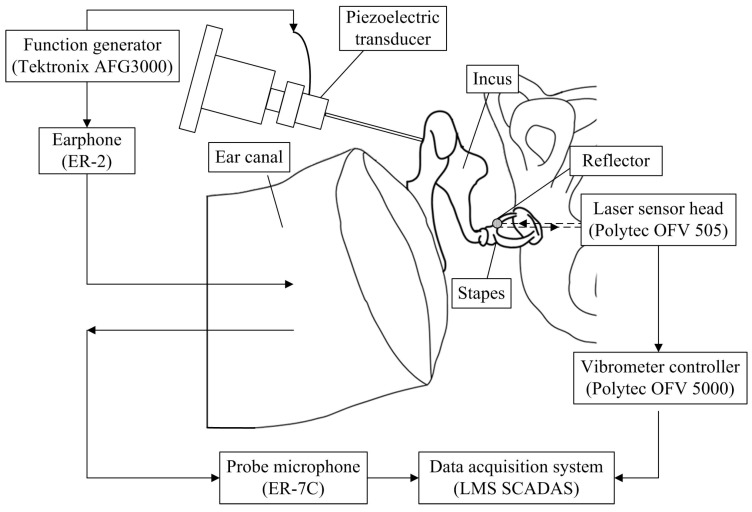
Experiment setup of the measurement system of the temporal bone experiment.

**Figure 3:**
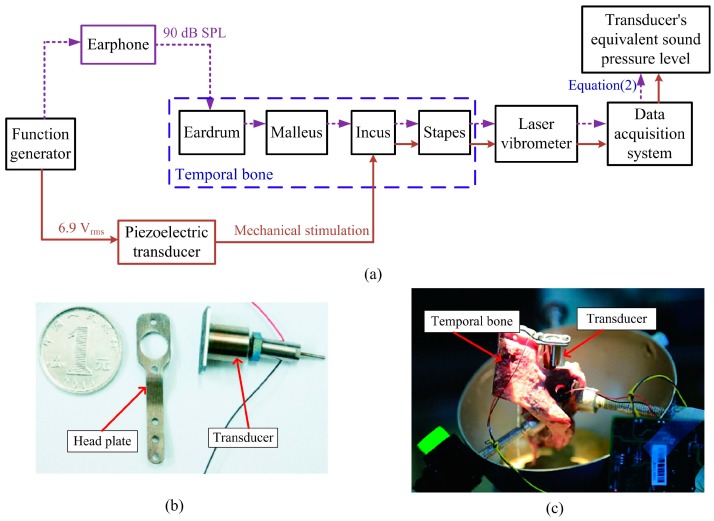
Flow diagram and pictures of the temporal bone experiment. (**a**) flow diagram of the temporal bone experiment (the solid arrow-lines indicate the acoustic stimulation testing, and the dashed arrow-lines indicate the transducer’s stimulation testing); (**b**) picture of the fabricated piezoelectric transducer; (**c**) a close view of the transducer implanted in the temporal bone.

To assess the hearing loss compensation capability of the transducer, a temporal bone experiment was conducted. A fresh human temporal bone was obtained from the Anatomy Research Department of Shanghai Medical College of Fudan University. The research was approved by the Ethical Committee at Zhongshan Hospital affiliated to Fudan University. Schematic of the temporal bone setup is shown in Figure 2, and the corresponding flow diagram of this experiment is illustrated in Figure 3a. For comparison, the normal middle ear’s response to acoustical outer ear canal stimulation was measured firstly (the dashed arrow-lines in Figure 3a). During this test, the level of the acoustic stimulation was set to 90 dB SPL since it is commonly used in related studies and gives high signal-to-noise ratio [22]. The temporal bone was held by a temporal bone holder, and the acoustic stimulation was applied to the eardrum by an earphone (ER-2, Etymotic Research, Elk Grove Village, IL, USA). The sound pressure of the stimulus sound was monitored by a probe microphone (ER-7C, Etymotic Research, Elk Grove Village, IL, USA) with its tip placed 1–2 mm in front of the eardrum. And the corresponding displacement of the stapes was measured by the laser vibrometer (OFV-505 sensor head, OFV-5000 vibrometer controller, Polytec, Waldbronn, Germany). To increase the reflection of the laser beam, we placed a small piece of reflective tape on the stapes.

After measuring the normal stapes displacement under acoustic stimulation, the corresponding stapes response excited by the transducer’s stimulation was tested (the solid arrow-lines in Figure 3a). The fabricated piezoelectric transducer (Figure 3b) was implanted in the temporal bone with its coupling rod’s tip attached to the incus body, as shown in Figure 3c. Then, a sine wave voltage produced by the function generator (AFG3000 series, Tektronix, Beaverton, OR, USA) was applied to the transducer, and the corresponding stapes displacement excited by the transducer was measured again by the laser vibrometer. To facilitate the assessment of the transducer's performance, the measured stapes displacement excited by the piezoelectric transducer was transformed to the equivalent sound pressure level ***P_eq_*** applied to the eardrum. This transformation was derived according to

(2) $$P_{eq}=90+20\times \log_{10}(\frac{d_{tr}}{d_{ac}})$$

where, ***d_tr_*** is the stapes displacement under transducer stimulus, and ***d_ac_*** is the stapes displacement under acoustical stimulus of 90 dB SPL.

### 2.5. Transducer Power Consumption

The power consumption of the implantable middle ear hearing device should be restricted since it is an implanted medical device. Thus, to ensure our proposed piezoelectric transducer is reasonable for implantable middle ear hearing device, the power consumption of the transducer was analyzed. When operating at frequencies far below its resonant frequency, piezoelectric transducer’s behavior approximates to that of a capacitor [22]. According to the manufacturer’s datasheet, the capacitance C of this piezoelectric stack is 25 nF. To simplify the estimation of the transducer’s power consumption, the influence of the external force can be neglected [27]. Experimental study showed that the difference between the actual power consumption and this estimated one was less than 3% [27], which meets the accuracy requirement of our research. Under this simplification, the piezoelectric transducer’s current *I_rms_* and the power consumption *P_rms_* can be calculated according to Equation (3) [7], when it is driven by a sinusoidal voltage *V_rms_* of a frequency *f*.

(3) $$I_{\mathrm{rms}}=2\pi fCV_{\mathrm{rms}},\;\mathrm{and}\;P_{\mathrm{rms}}=I_{\mathrm{rms}}V_{\mathrm{rms}}\cos\theta =2\pi fCV_{\mathrm{rms}}^{2}\cos\theta$$

where, cos *θ* is the power factor, and *θ* is phase of the piezoelectric transducer’s impedance.

## 3. Results and Discussion

### 3.1. Position Adjustments of the Coupling Rod’s Tip

**Figure 4:**
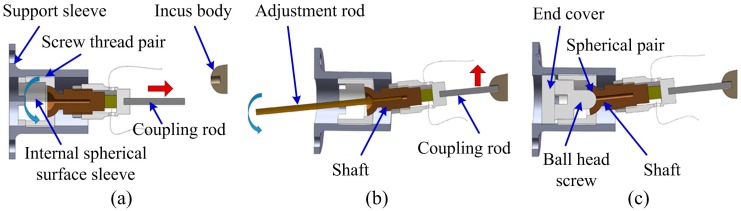
Position adjustments of the coupling rod’s tip. (**a**) Axial adjustment by screwing the internal spherical surface sleeve; (**b**) radial adjustment by rotating the shaft with an adjustment rod; (**c**) fix of the coupling rod by screwing the ball head screw and the end cover.

It is difficult to maintain the position of an implantable transducer by hand guidance, because both the mastoid and middle ear region of the skull are extraordinary small and have complex anatomical structures. Thus, a positioning system for the transducer that can be anchored to the skull is important for its implantation. The detailed adjusting process of our piezoelectric transducer is illustrated in Figure 4. First, the body of the incus was exposed surgically. Then, the head plate attached with the support sleeve was fixed onto the surface of the skull bone in an area which borders the mastoid antrum. After that, the tip of the transducer was advanced toward the incus body by turning the internal spherical surface sleeve, as shown in Figure 4a. Based on this step, the coupling rod’s tip can be freely positioned axially. When the coupling rod was turned close to the position of the incus body, put an adjusting rod into the transducer’s shaft and rotated it to adjust the radial position of the coupling rod’s tip (Figure 4b). After the coupling rod’s tip was adjusted in contact with the incus body, fixed the coupling rod in place by tightening the ball head screw and the end cover, respectively (Figure 4c).

The contact force between the coupling rod’s tip and the incus body is important for the performance of the transducer. Insufficient contact force on the incus body decreases the energy transfer, while too much contact force can induce a conductive loss. However, we have not designed a device to precisely control this force yet, for this preliminary study mainly focused on the evaluation of the new concept of the piezoelectric transducer. In above temporal bone experiment, we accomplished the attachment of the coupling rod to the incus body just based on a visual control of the surgeon. To precisely control the contact force, Jenkins et al. [28] designed a system called Transducer Loading Assistant (TLA), which works by measuring the change in the transducer’s electrical impedance and inductance. In our further studies, we will develop similar system for our transducer.

### 3.2. Vibration Displacement of the Piezoelectric Transducer

**Figure 5:**
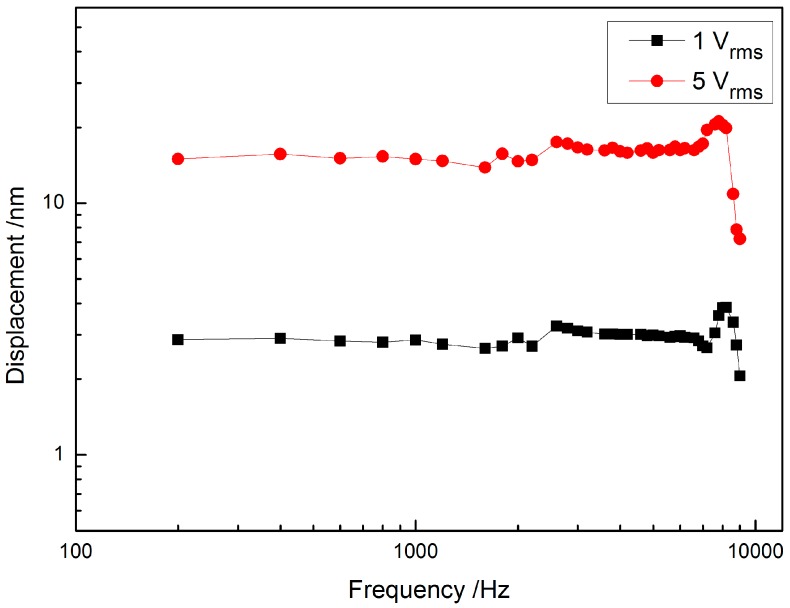
Frequency response of the piezoelectric transducer vibration at two applied voltage levels.

High frequency hearing loss is the most common type of sensorineural hearing loss [3]. Therefore, whether a transducer can perform efficiently at high frequency is important for the implantable middle ear hearing device. The measured displacement of the designed piezoelectric transducer is shown in Figure 5. It shows that our transducer has a flat frequency response up to 8 kHz, which is higher than the upper frequency limit (5.5 kHz) of a typical piezoelectric cantilever transducer [29]. Most hearing aids currently available on the market can only provide effective amplification for frequencies below 5 kHz [30,31]. Thus, our piezoelectric transducer has a wider bandwidth and meets the high-frequency-output’s requirement of the implantable middle ear hearing device. In addition, Figure 5 also demonstrates that the transducer’s vibration displacement is proportional to its driving voltage, that is, this piezoelectric transducer has a property of voltage-displacement linearity. This property will facilitate the development of the IMEHD’s signal processing algorithms.

**Figure 6:**
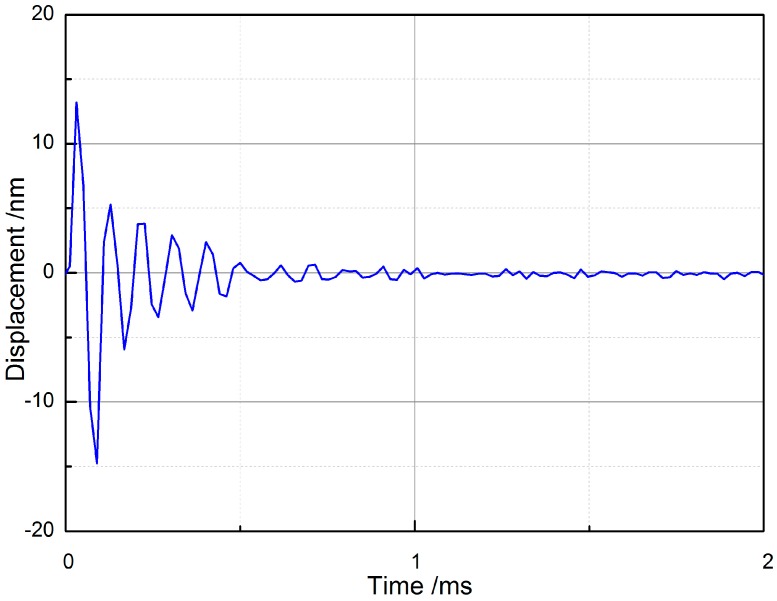
Pulse response of the piezoelectric transducer.

Apart from a larger bandwidth, the dynamic behavior in the time domain of an IMEHD’s transducer is vital important for its transfer quality [18]. Figure 6 shows the transient response of the piezoelectric transducer driven by a voltage pulse with a magnitude of 12 V_P-P_ and duration of 50 μs. The release time of the designed transducer is about 1 ms, which is much less than that value of conventional hearing aids (10 ms to several seconds [32]). Compared to another incus-body driving type implantable middle ear hearing device TICA, which has a release time of about 2 ms [33], our piezoelectric transducer still shows the advantage of fast response. Considering short release time offers greater speech recognition benefits [34], the application of our transducer can improve patients’ speech recognition.

### 3.3. Distortion Analysis of the Transducer

**Figure 7:**
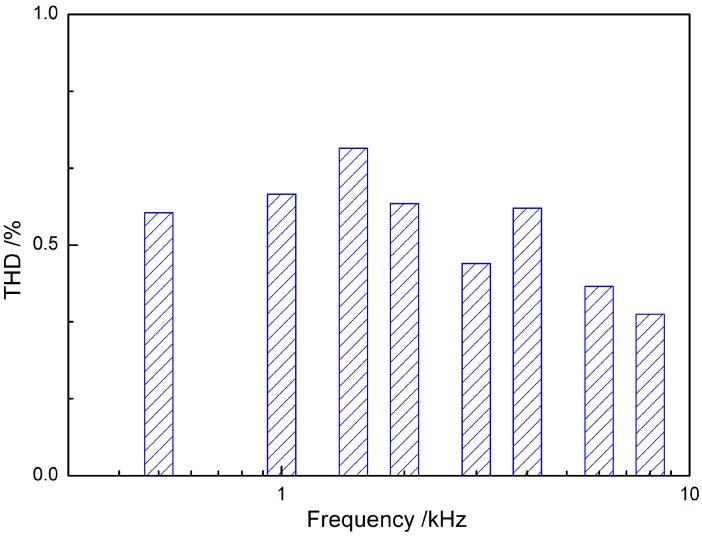
Histogram of the transducer’s total harmonic distortion (THD).

As stated before, low nonlinear distortions, which relates to the high-fidelity sound’s production, is important for patients’ speech and music perception [25]. The American National Standards Institute (ANSI) suggests using the total harmonic distortion (THD) to evaluate the distortion of the hearing aids [35]. A typical hearing aid’s THD lies between 2% and 3% [36]. Considering implantable middle ear hearing device is a kind of implantable hearing aid, many researchers also utilized the THD to assess their implantable middle ear hearing device’s sound quality [7,22,26]. The THD of our transducer is displayed in Figure 7, which has a maximum value of 0.71% at 1.5 kHz. This value is much less than that of hearing aids (2% and 3% [36]). Besides, the THD is less than 0.5% at high frequencies (>4 kHz). According to Fredrickson et al.’s report [37], their electromagnetic implantable middle ear hearing device, which is also an incus-body driving type IMEHD, reached values of between 0.5% and 1.1% (500 Hz to 10 kHz). Comparing with their electromagnetic transducer, our piezoelectric transducer still holds the advantage of lower THD, especially at high frequencies.

### 3.4. Temporal Bone Experiment

**Figure 8:**
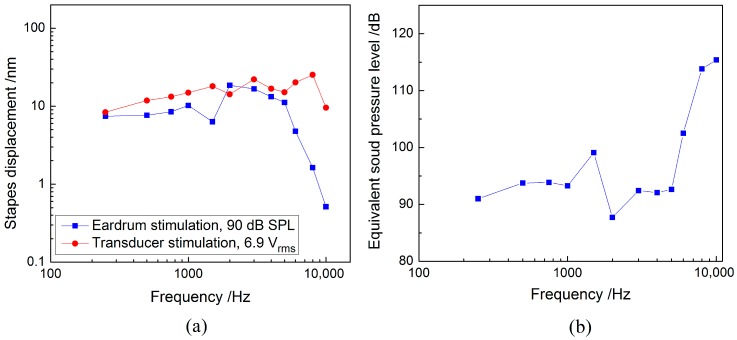
Temporal bone experimental results for the piezoelectric transducer: (**a**) comparison of stapes displacements driven by the piezoelectric transducer and a 90 dB SPL acoustic stimulation at the eardrum; (**b**) equivalent sound pressure levels at the eardrum for the piezoelectric transducer excitation at 6.9 V_rms_.

The measured stapes displacements driven by the piezoelectric transducer and the acoustic stimulation were shown in Figure 8a. It shows that the stapes displacement from transducer excitation at 6.9 V_rms_ is similar to that from acoustical stimulation at 90 dB SPL at low frequencies. At high frequencies (above 5 kHz), the stapes displacement excited by the transducer is considerably larger than that from the acoustic stimulation. Figure 8b displays the deduced equivalent sound pressure level of this transducer’s stimulation. As shown in Figure 8b, the transducer driven by 6.9 V_rms_ can generate about 90 dB SPL equivalent sound pressure at the eardrum below 5 kHz, and more than 100 dB SPL at higher frequencies (above 5 kHz). Thus, this piezoelectric transducer can compensate severe hearing impairment at high frequencies. Given that most sensorineural hearing loss is severe at higher frequencies [3], this transducer’s better high-frequency capability is a valuable aspect of its application in IMEHDs.

It should be noted that the stapes displacement of our temporal bone (10.24 nm at 1 kHz for 90 dB SPL applied at the eardrum) is relatively small compared with the reported average value (about 30 nm at 1 kHz for 90 dB SPL applied at the eardrum) [38]. This may be attributed to the individual temporal bone difference in geometry and material properties, for individual anatomical and physiologic differences of the external ear and middle ear can induce up to 25 dB individual variations in hearing thresholds [39]. Besides, in GAN et al.’s fresh temporal bones experimental study [40], 2 out of 9 temporal bones also had stapes displacements close to 10 nm (at 1 kHz under 90 dB SPL). Thus, the deviation of our temporal bone’s stapes displacement to the average value is acceptable. In addition, since comparison are made within the same temporal bone and the stiffness’ enlargement of the temporal bone has a similar influence on the transducer’s stimulation and the sound stimulation, above deviation does not affect the validity of our study, which focused on comparing these two simulations’ response.

### 3.5. Transducer Power Consumption

The fabricated piezoelectric-stack transducer has a capacitance of 25 nF, and an impedance phase of about −89.2°, which resulted in an root-mean-square current consumption of 0.157 mA and the power consumption of about 2.19 μW per volt of excitation at 1 kHz. Using this transducer to produce a 100 dB SPL equivalent sound pressure at 1 kHz, the power consumption is about 0.49 mW.

According to Leysieffer’s report [33], Ball’s invented electromagnetic transducers has a power consumption of about 1.5 W to produce a 100 dB SPL equivalent sound pressure at 1 kHz [41]. In comparison with Ball’s transducer, the corresponding power consumption of our piezoelectric transducer, which is 0.49 mW, is much small. To stimulate the same level of equivalent sound pressure level at 1 kHz, Maniglia et al. [42] proposed contactless electromagnetic transducer showed a power consumption of about 10 mW, which is still larger than that of our transducer (0.49 mW). Thus, the power consumption of our piezoelectric transducer is less than that of electromagnetic hearing implants and reasonable for implantable hearing aids. Besides, power consumption of our transducer can be further reduced by incorporating a flextensional amplifier for its piezoelectric stack [43].

## 4. Conclusions

In this paper, we proposed and designed a new piezoelectric transducer for the incus-body driving-type implantable middle ear hearing device, which has the benefits of minor damage to the ossicular chain and less side effect on patients’ high-frequency residual hearing. This transducer is based on a piezoelectric stack, with the advantages of ease of fabrication and compatibility with external magnetic fields. The bench testing demonstrated that the transducer’s prototype has a wide bandwidth, short release time, and low distortion. It has a power consumption of 2.19 μW per volt of excitation at 1 kHz, which is reasonable for implantable middle ear hearing device. The temporal bone experimental results showed that the transducer could stimulate the ossicular chain at frequencies and magnitudes appropriate for treating sensorineural hearing loss. It could successfully transmit high-quality sound through mechanical stimulation of the incus body. Moreover, it performs better at high frequencies which is a valuable aspect of its performance, given that the most commonly encountered pattern of sensorineural hearing loss is severe at high frequencies.

Although the present study demonstrates that this piezoelectric transducer is a viable concept for implantable middle ear hearing devices, further work is still necessary to improve the adjusting system for controlling the pre-load between the transducer’s coupling rod tip and the incus body, and optimize the transducer design. In addition, continued cadaveric and animal testing should be carried out in the future.
